# A role for NT-3 in the hyperinnervation of neonatally wounded skin

**DOI:** 10.1016/j.pain.2012.07.012

**Published:** 2012-10

**Authors:** Simon Beggs, Debie Alvares, Andrew Moss, Gillian Currie, Jacqueta Middleton, Michael W. Salter, Maria Fitzgerald

**Affiliations:** a Programme in Neurosciences & Mental Health, The Hospital for Sick Children, Toronto, ON, Canada; b Faculty of Dentistry, University of Toronto, Toronto, ON, Canada; c Department of Neuroscience, Physiology & Pharmacology, University College London, London, UK

**Keywords:** Neurotrophin, Pain, Sprouting, Sensory neuron, Cutaneous

## Abstract

Neurotrophin-3 (NT-3) is a target-derived neurotrophic factor that regulates sensory neuronal survival and growth. Here we report that NT-3 plays a critical permissive role in cutaneous sensory nerve sprouting that contributes to pain and sensitivity following skin wounding in young animals. Sensory terminal sprouting in neonatally wounded dermis and epidermis is accompanied by increased NT-3 transcription, NT-3 protein levels, and NT-3 protein release 3-7 days post skin injury in newborn rats and mice. Functional blockade of NT-3 activity with specific antibodies greatly reduces sensory neurite outgrowth induced by wounded skin, but not by naïve skin, in dorsal root ganglion/skin co-cultures. The requirement for NT-3 for sensory terminal sprouting in vivo is confirmed by the absence of wound-induced hyperinnervation in heterozygous transgenic mice (NT-3^+/−^lacZ). We conclude that upregulation of NT-3 in neonatally wounded skin is a critical factor mediating the sensory nerve sprouting that underlies hypersensitivity and pain following skin injury.

## TOC summary

Newborn skin wounding causes an upregulation of NT-3 and cutaneous sensory hyperinnervation that contributes to the increased pain and sensitivity associated with wounding.

## 1 Introduction

The development of sensory innervation of the skin is highly dependent upon target-derived neurotrophins [43] that control the survival, development, and function of peripheral and central neurons [27]. Each neurotrophin: nerve growth factor (NGF), brain-derived neurotrophic factor, neurotrophin-3 (NT-3), and neurotrophin-4 has a characteristic spatial and temporal expression pattern and specificity throughout development, acting through receptors TrkA, TrkB, and TrkC [32,47]. In the developing peripheral somatosensory system, limiting quantities of neurotrophins control the number of surviving sensory neurons and the target innervation density [16,27]. Synthesis of NGF in the developing epithelium begins with the arrival of sensory fibres [15], and its concentration in the skin is directly correlated with the final innervation density [23], particularly of nociceptors [2,27,43], independent of its role in sensory neuron survival [14]. While NT-3 is classically associated with the survival of proprioceptors and the sensory innervation of joints and muscle [10], it is also produced by keratinocytes [38] and skin fibroblasts [29] in vitro and plays a role in skin innervation and mechanoreceptor sensory neuron survival [1,20]. In the skin, TrkA-expressing cutaneous nociceptor terminals are NGF-sensitive, while TrkC-expressing mechanoreceptors are NT-3-sensitive. However, the importance of NT-3 in determining skin innervation density is less clear than for NGF. Exposure to a localized source of NT-3 induces attractive growth cone turning via activation of specific growth proteins in vivo and in vitro [39]. In addition, NT-3 reduces overlap between sensory dermatomal boundaries in adulthood [50].

NT-3 levels in adult skin increase following tissue damage, such as skin irritation or diabetic neuropathy [30,51], but little is known about the extent and pattern of injury-induced NT-3 upregulation in the skin. Functional blockade of NT-3 prevents reinnervation by the spiral ganglion nerve following cochlear damage in vitro [58], but nothing is known of the role of NT-3 upon cutaneous sensory sprouting following skin damage. Here we test the hypothesis that NT-3 plays a key role in regulating cutaneous innervation following skin damage in early postnatal life.

Skin wounding is accompanied by sprouting of sensory nerves into the damaged tissue that contributes to both pain and sensitivity [25,31,40,53]. This response is especially marked in young animals, where skin wounding at birth causes a striking and prolonged hyperinnervation in and around the wounded site by both A- and C-fibre sensory axons [4,23,24,35,36,48,49], accompanied by behavioural sensitivity [4] and prolonged central sensitization in the dorsal horn in later life [54]. This sprouting may contribute to the prolonged changes in central sensory processing that are triggered by infant pain and injury [19]. Skin wounding is accompanied by a marked upregulation of NGF, particularly in young rats [13], but NGF alone is not responsible for triggering and maintaining subsequent sensory hyperinnervation [48]. Here we show that NT-3 is upregulated in the injured skin of young rats and mice and is accompanied by hyperinnervation of the wounded skin. Functional blockade of NT-3 activity in vitro and in vivo prevents this hyperinnervation, showing that NT-3 has a critical permissive role in this injury response.

## 2 Methods

### 2.1 Neonatal skin wounding

Newborn C67Bl/6 mouse pups or Sprague-Dawley rat pups were anaesthetised with halothane/oxygen mixture and a full thickness 1-mm^2^ flap of skin was removed from the dorsal surface of one hind paw under sterile conditions. Following recovery, pups were returned to their litters. Three, 5, or 7 days after wounding, pups were terminally anaesthetised and samples taken for analysis of cutaneous innervation, protein levels, or co-culture experiments.

### 2.2 Co-cultures

Co-cultures of skin and dorsal root ganglia (DRG) in collagen were prepared as previously described [48]. Briefly, lumbar DRG were dissected from newborn (P0) rat pups and subdivided into 4 explants. Each explant was positioned 1.5-2 mm from a piece of dorsal hind paw skin (25 mm^2^, naive or P0 wound + 3 days) and embedded in 30 μL of prepared rat tail collagen. Once set, the co-cultures were cultured in 2.5 mL of Ham’s nutrient mixture F14 plus 0.5% l-glutamine (Gibco; Life Technologies, Grand Island, NY, USA), 1% penicillin-streptomycin (Gibco), and 2% Ultroser G (Gibco) for 24 hours at 36°C in 5% CO_2_. For the antibody-blocking experiments, a function-blocking anti-NT-3 antibody (2 ng/mL) was added for the duration of the experiment.

The following combinations were prepared with P0 DRG: P3 normal skin; P0 wounded + 3 days skin; P3 normal skin + 2 ng/mL anti-NT-3; P0 wound + 3 skin + 2 ng/mL anti-NT-3. For each condition, n = 4 explants were used from 4 rats (16 total).

After 24 hours, the co-cultures were washed, fixed in 4% paraformaldehyde for 24 hours, incubated in blocking solution (phosphate-buffered saline [PBS] + 0.3% triton X-100 + 10% normal donkey serum) for 1 hour and then overnight at room temperature with anti-PGP 9.5 (1:1000, Ultraclone; Lucigen Corporation, Middleton, WI, USA), followed by Vectastain ABC kit (Vector Laboratories, Burlingame, CA, USA) and DAB 3,3’-Diamonobenzidine colour reaction. Neurite outgrowth was analysed by counting the number of processes extending from the DRG explants in the quadrant adjacent to the skin. Results were then expressed as the mean number of neurites ± SEM and statistically analyzed by one-way analysis of variance.

### 2.3 ELISA

Enzyme-linked immunosorbent assays (ELISAs; Chemicon; Merck Millipore, Billerica, MA, USA) were undertaken to quantify (1) NT-3 protein levels in wounded skin and (2) the amount of NT-3 protein secreted by the wounded skin.

To quantify NT-3 protein levels in the skin, samples were taken 3 days postwounding from skin surrounding the wound (up to 1 mm away) and from equivalent normal P3 skin, weighed, and freeze-dried in sterile cryotubes. Samples were homogenised, centrifuged at 13,000 rpm at 4°C for 1 hour and supernatants collected for ELISA analysis (n = 6 samples from separate animals for each group).

To quantify the amount of NT-3 secreted from wounded skin, skin samples were taken immediately post wounding and maintained in culture for 24 hours. Unwounded tissue from the contralateral side was used for comparison (n = 6 animals). Culture conditions were the same as that described above. After 24 hours, culture medium was taken and NT-3 measured by ELISA. Results were analysed by paired *t*-test.

### 2.4 NT-3-deficient mice

Mice deficient in NT-3 were obtained from the Mutant Mouse Regional Resource Centre, (MMRRC:000191, stock Ntf3 < tm1Lfr >), where the entire protein coding region of NT-3 was replaced with lacZ [17], creating the null mutation NT-3^lacZneo^. As homozygous null mice die perinatally [56], heterozygotes (NT-3^lacZneo^/+) were used here. NT-3 gene expression and promoter activity was characterized by detecting lacZ activity in heterozygous NT-3^lacZneo^ mice in which one allele drives β-galactosidase (β-gal) under the control of the NT-3 promoter. A full-thickness piece of skin was removed from the dorsal surface of the hind paw of anaesthetized NT-3^lacZneo^/+ mice as described above. Skin wounding was performed blind and genotype revealed by subsequent β-gal expression. After 7 days, mice were terminally anaesthetized, perfused with 4% paraformaldehyde, and cryoprotected in 30% sucrose solution in 0.1 M PBS. Cryostat sections (50 μm) were made transversely through the hind paw and processed for lacZ staining as previously described (Vigers et al., 2000). Briefly, sections were washed in X-gal wash (0.1 M PBS, 2 mM MgCl_2_, 5 nM EGTA, 0.01% sodium deoxycholate, 0.02% Nonidet-P40) for 10 minutes and then stained overnight at 37°C in X-gal staining solution (X-gal wash plus 5 mM potassium ferricyanide, 5 mM potassium ferricyanide, 1 mg/mL 5-bromo-4-chloro-3-indolyl-β-galactopyranoside [X-gal, Invitrogen; Life Technologies]). Sections were then washed in distilled water and counterstained with neutral red for 1 minute. After staining, samples were rinsed in distilled water 3 times, dehydrated with ethanol (5 minutes each in 70%, 95%, and 100%), cleared in xylene, and mounted.

### 2.5 Skin immunostaining

Rat and mice pups were terminally anaesthetised and transcardially perfused with 4% paraformaldehyde. Frozen 50-μm sections through the wounded region of skin (rats) or entire hind paw (mice) and appropriate naive and contralateral controls were cut for subsequent immunohistochemistry. Sections were washed in 0.1 M PBS and then blocked in PBS with 10% normal donkey serum and 0.3% Triton-X for 1 hour. Serial sections were then incubated in primary antibodies (PGP 9.5 [1:500; Dako, Glostrup, Denmark], calcitonin gene-related peptide (CGRP; 1:2000; Sigma-Aldrich, St. Louis, MO, USA), Type IV Collagen (1:2000; SouthernBiotech, Birmingham, AL, USA), and P2X3 (1:500; Neuromics, Edina, MN, USA) overnight at room temperature. Staining was then visualized using appropriate fluorescently conjugated secondary antibodies (1:1000; Jackson, West Grove, PA, USA) and sections mounted.

Fluorescent images were captured with either a conventional fluorescent microscope or with a Zeiss Axiovert confocal microscope (Carl Zeiss, Cambridge, UK) using Volocity software (Perkin Elmer, Waltham, MA, USA). Confocal images of 0.5 μm z-steps were taken through each section. Images were then deconvolved and the fluorescence volume quantified using Volocity software. The results were then compared by one-way analysis of variance (Graphpad Prism, La Jolla, CA, USA).

## 3 Results

### 3.1 Cutaneous afferents hyperinnervate neonatally wounded skin

**Fig. 1:**
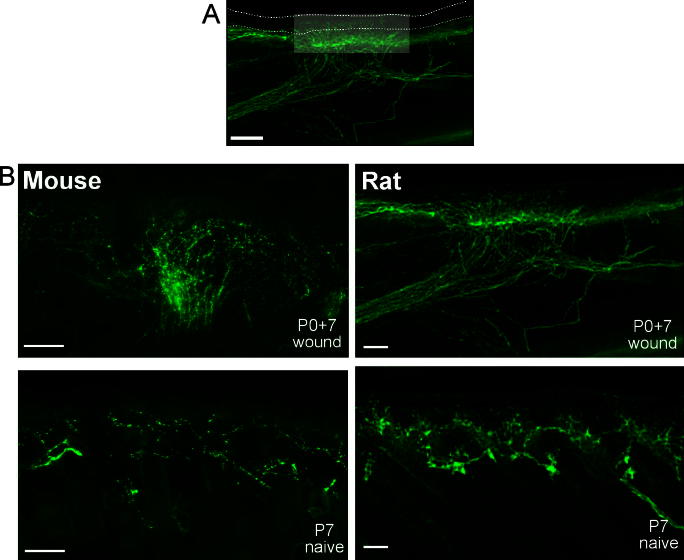
Neonatal wounding induces skin hyperinnervation in rat and mouse. (A) PGP9.5 immunohistochemistry in rat skin 7 days following P0 full-thickness skin wounding. The lightened area represents the extent of the skin wound. Dotted lines indicate the surface of the epidermis (upper) and epidermal/dermal boundary. Scale bar represents 200 μm. (B) PGP9.5 immunolabeling of cutaneous nerve fibres in mouse (left) and rat (right). Upper panels show cutaneous hyperinnervation 7 days following P0 wound. Lower panels show P7 naïve skin innervation. Scale bars represent 100 μm.

Primary afferents innervating the skin of mouse and rat were revealed immunohistochemically using the pan-neuronal marker PGP9.5. A full-thickness skin flap was removed from the dorsal surface of one hind paw in newborn (P0) rat (Sprague-Dawley) and mouse (C57Bl/6) pups under anaesthesia (extent of wound is indicated by the highlighted area on the transverse skin section shown in Fig. 1A). Seven days after wounding, cutaneous innervation in the region of the wound was examined and compared with postnatal day 7 (P7) naive animals (Fig. 1B). Despite near-complete healing, neonatal wounding had induced a pronounced hyperinnervation of the skin, as indicated by the dense network of PGP9.5 immunoreactive fibres in both mouse (Fig. 1B, top left) and rat (Fig. 1B, top right) compared to age-matched littermate controls (Fig. 1B, lower panels).

### 3.2 Neurotrophin 3 is increased in and secreted from wounded skin

**Fig. 2:**
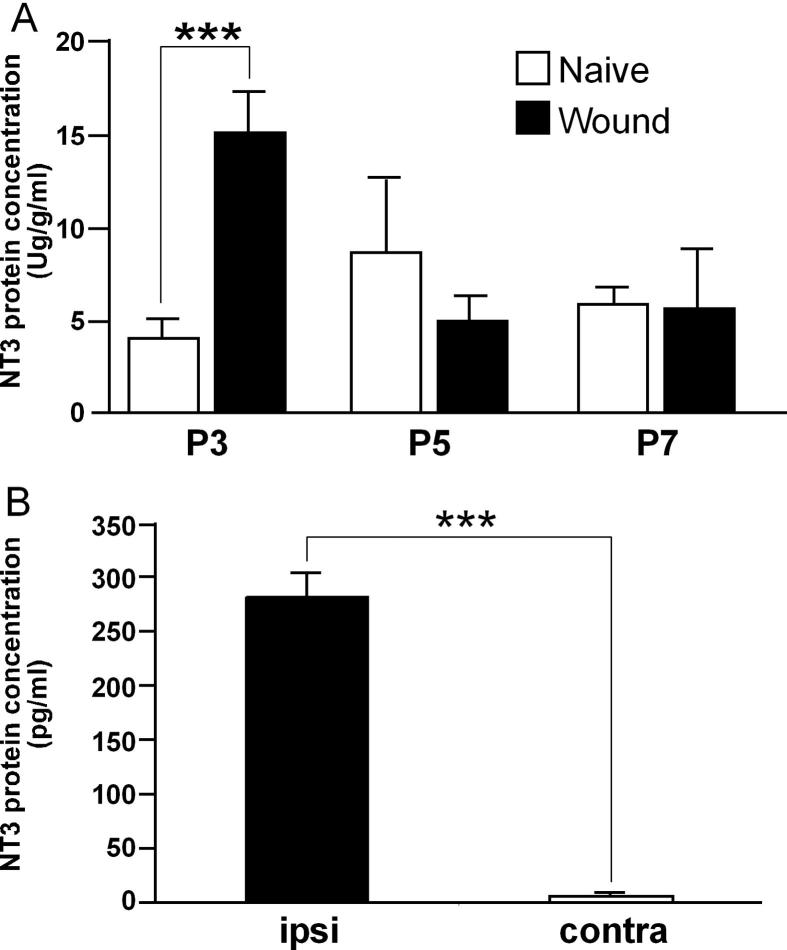
Neonatal wounding induces the production and secretion of neurotrophin-3 (NT-3) in skin. (A) Enzyme-linked immunosorbent assay (ELISA) of NT-3 protein concentration in rat skin at the indicated times following P0 skin wounding (n = 6-8 for each age and condition). ^∗∗^*P* < 0.001 (one-way analysis of variance). Data are represented as mean ± SEM. (B) ELISA of NT-3 protein levels secreted from P0 + 3 wounded or contralateral skin (n = 6 for each) following 24 hours in culture. ^∗∗∗^*P* < 0001 (*t*-test). Data are represented as mean ± SEM.

To investigate the potential neurotrophin dependency of skin hyperinnervation, NT-3 protein levels in skin samples were measured by ELISA at 3, 5, and 7 days following P0 wounding and compared to naïve age-matched littermate controls (Fig. 2A). We found a significant increase in skin NT-3 protein 3 days after P0 skin wounding (*P* < 0.001). Levels then decreased to naïve values by 5 and 7 days. There was no change in naïve skin NT-3 protein concentration across the first postnatal week.

We then investigated whether this increased NT-3 protein was secreted by wounded skin. Wounded and contralateral intact skin samples were taken from the same rat pup and grown in culture for 24 hours. Fig. 2B shows that there is a marked increase in NT-3 protein in the culture medium of wounded skin, showing that NT-3 secretion from the wounded skin is significantly greater than control tissue (Fig. 2B; *P* < 0.0001).

### 3.3 NT-3 secreted from wounded skin stimulates sensory-afferent neurite outgrowth

**Fig. 3:**
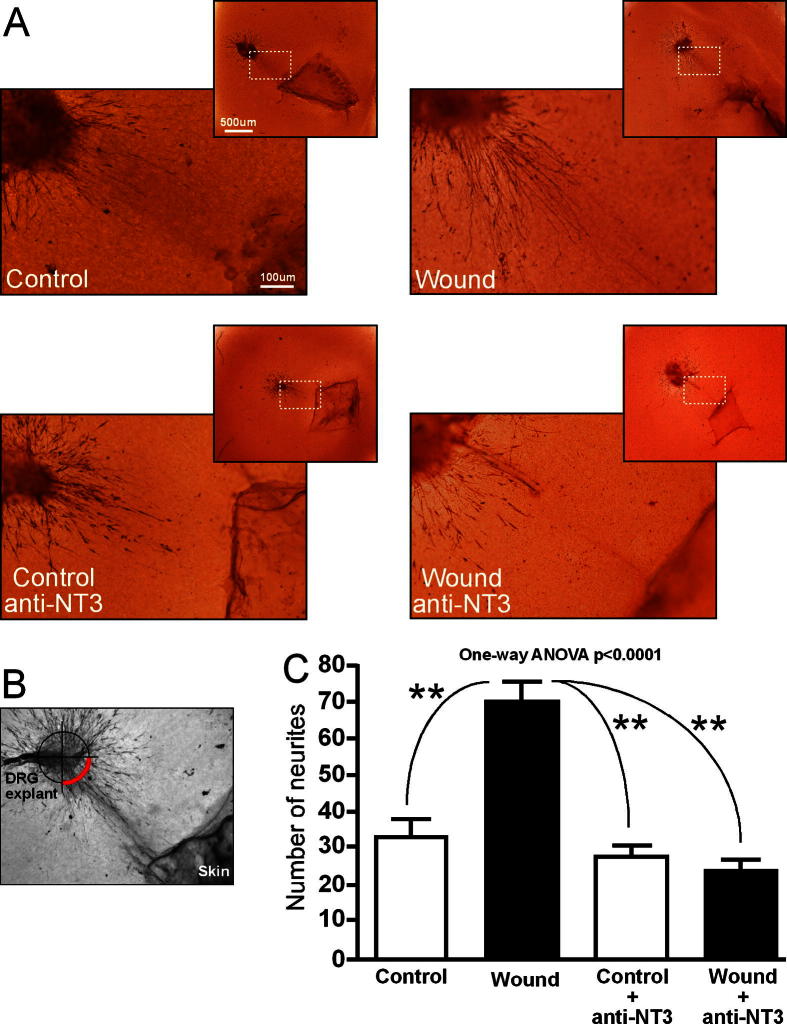
Neurotrophin-3 (NT-3) released from wounded skin selectively stimulates primary sensory neurite outgrowth. (A) Neurite outgrowth of newborn dorsal root ganglia (DRG) explants co-cultured for 24 hours with P3-naïve or P0 + 3-wounded skin (upper panels) and in the presence of a function blocking NT-3 antibody (lower panels). Scale bar represents 100 μm for higher-power images and 500 μm for lower-power images. (B) Quantification method for measuring neurite outgrowth. Red quadrant indicates the region within which neurite crossings were counted. (C) Number of DRG neurites growing towards skin explant (mean of 16 explants from 4 rats per condition). *P* < 0.0001 (one-way analysis of variance [ANOVA], Newman-Keuls multiple comparison test).

To investigate the functional significance of the increased production and secretion of NT-3 by wounded skin, we assayed neurite outgrowth from a neonatal DRG explant co-cultured with control (P3) or wounded (P0 wound + 3 days) skin (Fig. 3). In order to measure the specific contribution of NT-3, a function-blocking NT-3 antibody was added to the co-cultures. Co-cultures were maintained for 24 hours, fixed, and neurite outgrowth revealed by PGP9.5 immunohistochemistry. There was significantly greater neurite outgrowth from the DRG explant towards the wounded skin compared to control skin (Fig. 3A top left, right), as quantified by counting the numbers of neurites in the quadrant adjacent to the skin (Fig. 3B). Addition of a function-blocking anti-NT-3 antibody abolished the trophic activity of the wounded skin, such that neurite outgrowth was not significantly different from control levels (Fig. 3C).

### 3.4 Neonatal skin wounding increases endogenous NT-3 promoter activity

Following the demonstration that neonatal skin secretes NT-3 following wounding, we assessed NT-3 promoter activity in NT-3^lacZneo^ mice. Because homozygous NT-3^lacZneo^ mice die at birth (Vigers et al., 2000), we used neonatal NT-3^lacZneo^/+ mice for all in vivo experiments. β-gal activity, as revealed by X-gal, allowed us to map the sites of NT-3 promoter activity. Eosin counterstaining showed no structural abnormalities within the paw or skin of NT-3-deficient mice.

**Fig. 4:**
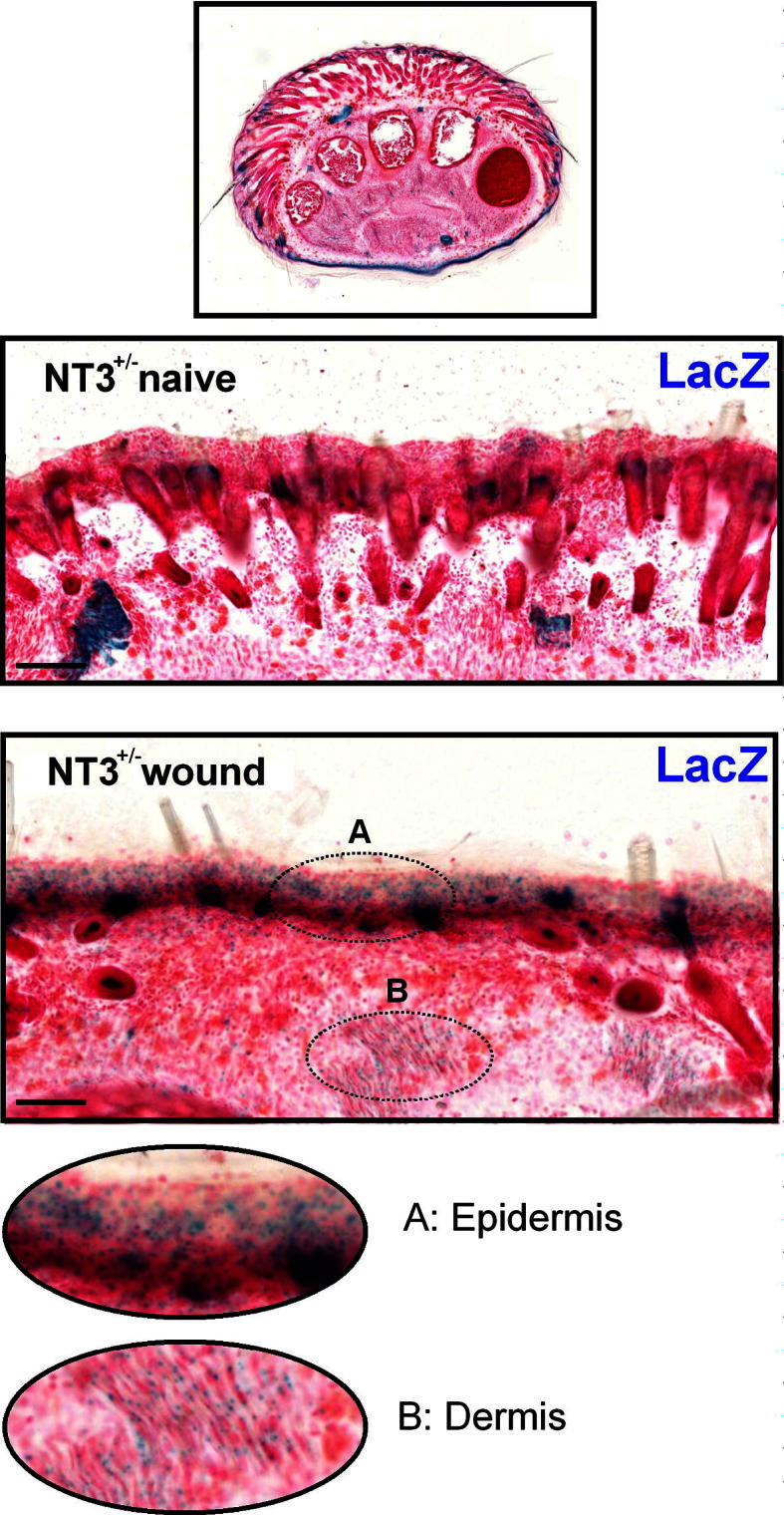
Skin wounding leads to *de novo* lacZ expression in dermis and epidermis in NT-3^lacZneo^/+ mice. LacZ expression (blue) in skin from NT-3^lacZneo^/+ mice. P7 naïve expression (upper panel) and 7 days following P0 skin wounds (lower panel). (A, B) Higher power areas of epidermal and dermal LacZ expression. NT3 (NT-3) = neurotrophin-3.

Fig. 4 shows a section through the complete paw of a naive P7 lacZ mouse. Higher power images of the skin in transverse section shows the distribution of β-gal in naive skin with sparse expression evident in hair follicles. In contrast, skin sections taken 7 days after P0 skin wounding show a marked β-gal expression in both the epidermis and deeper dermis (Fig. 4A, B), indicating *de novo* NT-3 promoter activity induced by skin wounding.

### 3.5 Hyperinnervation is absent in NT-3-deficient mice

**Fig. 5:**
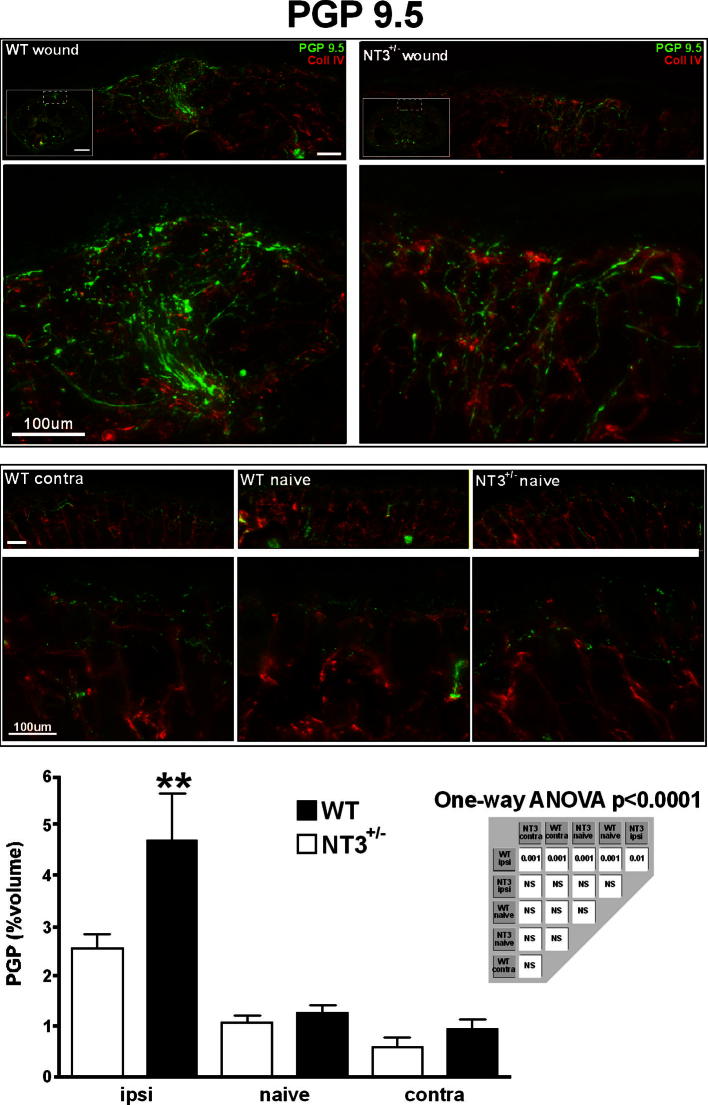
Absence of hyperinnervation in neurotrophin-3 (NT-3)-deficient skin following wounding. Comparison of wound-induced skin hyperinnervation in wild-type (left) and NT-3^lacZneo^/+ mice (right). Cutaneous innervation is shown by PGP9.5 immunoreactivity (green) (n = 10 for each genotype and condition). *P* < 0.0001 (one-way analysis of variance [ANOVA], Newman-Keuls multiple-comparison test). Scale bar = 100 μm.

We next examined whether reducing NT-3 in vivo would attenuate wound-induced hyperinnervation. As NT-3^lacZneo^/+ mice have one allele driving β-gal under the control of the NT-3 promoter, they correspondingly produce less NT-3 with increased promoter activity. To see whether attenuated NT-3 production affected skin hyperinnervation following neonatal skin wounding, skin sections from P0 wounded + 7 NT-3^lacZneo^/+ and wild-type age-matched littermate controls were labelled with the pan-neuronal marker PGP9.5 (Fig. 5; c.f. Fig. 1). Wounding of wild-type mice resulted in a characteristic hyperinnervation of the skin 7 days later (Fig. 5, top left panels). In contrast, wounding of NT-3^lacZneo^/+ led to a greatly diminished response (Fig. 5, top right panels). Analysis of PGP9.5 immunofluorescence within the skin sections showed that, while in wild-type mice there a highly significant increase in PGP9.5 immunoreactivity 7 days following neonatal skin wounding (Fig. 5; *P* < 0.001), wounding of NT-3^lacZneo^/+ skin resulted in innervation density that was not statistically different from age-matched naive animals (Fig. 5 graph). Innervation of wild-type naive, wild-type contra, and NT-3^lacZneo^/+ naive skin were not statistically different (as shown by PGP9.5 immunoreactivity).

### 3.6 Peptidergic afferents preferentially hyperinnervate neonatally wounded skin

**Fig. 6:**
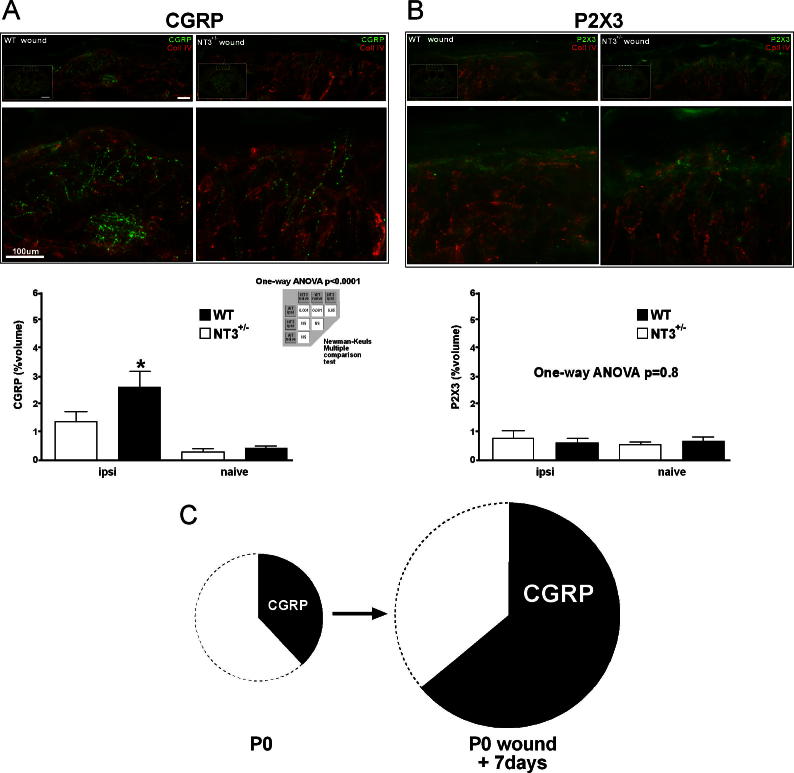
Selective hyperinnervation of neonatally wounded skin by peptidergic afferents. Hyperinnervation of neonatally wounded skin by peptidergic afferents (A: calcitonin gene-related peptide [CGRP]) but not by nonpeptidergic afferents (B: P2X3 right); n = 10 for each condition and genotype. *P* < 0.001 (one-way analysis of variance, Newman-Keuls multiple-comparison test. (C) Pie chart representing the relative contribution of CGRP to the entire cutaneous innervation. WT = wild-type; NT3 = neurotrophin-3.

Having shown that hyperinnervation following skin wounding is NT-3 dependent, we wished to establish which populations of cutaneous afferents are sensitive to levels of NT-3 under conditions of skin wounding in vivo. To test this, we selectively immunolabeled peptidergic and nonpeptidergic primary afferents using antibodies to CGRP (Fig. 6A) and P2X3 (Fig. 6B), respectively. In wild-type mice there was robust CGRP+ve fibre sprouting (Fig. 6A, left panels), which was dramatically reduced in NT-3^lacZneo^/+ mice (Fig. 6A, right panels) such that levels were not significantly different from wild-type controls (Fig. 6A, graph). There was no significant difference between naive wild-type and NT-3^lacZneo^/+ controls. Interestingly, no contribution to hyperinnervation following wounding was made from P2X3+ve nonpeptidergic C fibres, with neither wild-type nor NT-3^lacZneo^/+-wounded skin being significantly different from naive age-matched controls (Fig. 6B). Thus, the phenotypic profile of hyperinnervated skin is shifted, compared to normal skin, towards a high proportion of CGRP+ve fibres (Fig. 6C), suggesting that these fibres are preferentially sensitive to increased NT-3 levels.

## 4 Discussion

Here we have shown that NT-3 protein is upregulated and secreted from neonatally wounded skin and that this NT-3 stimulates growth of peripheral sensory nerve terminals, resulting in hyperinnervation of the wounded area. NT-3 protein levels in target tissues are normally very low, in the order of 10-100 ng/g protein [37], and the 3-fold upregulation in the skin and the striking 25-fold increase in release is likely to be highly biologically significant. During normal development, NT-3 mRNA is expressed in embryonic and neonatal dermis and epithelium [52] and plays a key role in the survival, growth, and target innervation pattern of cutaneous sensory neurons [43]. The role of NT-3 in the biology of the skin has not been fully elucidated, but it is well established that null mutants lack D hair afferents and slowly adapting mechanoreceptors and their associated end organs [1], while NT-3-overexpressing mice have increased unmyelinated and small myelinated axons within the epidermis and dermis [3,41] and altered skin innervation patterns [34,50]. NT-3 continues to be expressed in adult skin at low levels, and while it is not known whether it is required for maintenance of normal innervation, it is notably upregulated in pathological states such as allergic skin diseases and in diabetic neuropathy in the adult [30,46]. Here we show for the first time that NT-3 is highly regulated in normal skin by mechanical skin wounding in the newborn period.

The source of NT-3 in our experiments could be skin keratinocytes, fibroblasts, melanocytes, or mast cells [42,46] enhanced by the heaping up of adjacent epidermis above the damaged area [40]. Our results are consistent with reports that NT-3-producing fibroblasts stimulate neurite outgrowth of sensory neurons in culture, mimicking the effect of skin-derived cells [29]. They also agree with the finding that functional blockade of NGF does not affect neonatal skin wound-induced neurite outgrowth [48], and also that NGF-dependent sensory neurons display repulsive behaviour towards each other in vitro, while NT-3-responsive neurites intermingle [44]. NT-3 is a mitogen for macrophages, and phagocytic activity may rise in wounded skin to allow removal of debris and encourage healing [33]. It also regulates mast cell maturation and numbers in neonatal skin [42,46]. In the lacZ skin sections, it is evident that NT-3 transcription occurs in the epidermis, but since it is secreted, its site of action could be elsewhere in the skin. NT-3 is also expressed in some sensory neurons in the embryo [52] and a percentage of large-diameter afferents in the skin in the adult [59], and this is confirmed in this study by galactosidase staining in dermal nerve bundles in lacZ mice.

NT-3 function in stimulating sensory nerve sprouting after neonatal wounding is likely to be a result of binding to TrkC receptors on the nerve terminals and anterograde Trk transport to the soma, enhanced by the sorting receptor, sortilin [47,55]. TrkC is a dependence receptor that instructs sensory neurons to die during development if not activated by NT-3 in embryonic life [45], but importantly, NT-3 can also activate TrkA and TrkB [35,47], sustaining the survival of many subsets of sensory neurons and promoting the formation of all types of sensory endings [20] and stimulating keratinocyte and hair follicle proliferation [9]. Trk signalling is more complex than previously thought involving several receptor subdomains, and there are “hot spots” on the TrkA receptor for binding of NT-3 as a heterologous ligand [28]. This is particularly relevant here, as the majority of sprouting afferents in wounded skin express CGRP, normally thought to express TrkA rather than TrkC. The low-affinity interactions of NT-3 with TrkA and TrkB requires high concentrations of the neurotrophin, and this may explain the dramatic decrease in innervation following neonatal skin wounding in heterozygous NT-3 mutant mice. Since effective signalling by neurotrophins results from the interaction between neurotrophins, their precursor proneurotrophins, p75NTR and Trks, and in addition, activation of Trks can occur through neurotrophin-independent mechanisms [52], no assumptions about Trk signalling in the sprouting nerve fibres can be made from these data. Furthermore, the skin wound may increase the growth capacity of sensory neurons through upregulation of numerous other related growth-associated genes [26].

One possibility is that NT-3 effects upon sprouting are mediated via stimulation of nonneuronal cells [42]. Interactions between neurotrophins and cytokines are likely to play an important role in the neural response to tissue damage: cytokines increase NT-3-stimulated nerve growth in vitro [21], and increase NT-3 expression when injected intradermally in vivo [8]. Immune activation is a requirement of NT-3-induced axonal sprouting in the central nervous system [11], and it is possible that this is also true following skin wounding.

Newborn mice and rats were used for this series of experiments and are born at a relatively earlier stage of development than humans. It has been estimated that mice and rats reach an equivalent stage of development as a human full-term infant by P7 [6,12,18], and as such, provide a postnatal animal model for infant pain mechanisms and, in particular, preterm infants, who are the most likely to receive necessary but potentially tissue-damaging procedures in neonatal intensive care [22,57] Recently there has been considerable interest in the prolonged consequences of surgery and intensive care in human infants [5,19], and there is evidence that early skin damage can have long-term effects on central sensory synaptic organisation [54] and upon responses to repeat wounding in later life [7]. NT-3 is only one member of a number of families of molecules that may be regulated by tissue damage and lead to sensory outgrowth, but these results show that it has a key role in the prolonged hyperinnervation that follows skin wounding in young rodents. Prevention of local NT-3 upregulation and the resulting hyperinnervation may be an important step towards reducing the effects of infant tissue trauma following surgery or intensive care.

## Conflict of interest statement

The authors declare no conflict of interest.
